# Body composition estimation from mobile phone three-dimensional imaging: evaluation of the USA army one-site method

**DOI:** 10.1017/S0007114524002216

**Published:** 2024-11-14

**Authors:** Christine M. Florez, Christian Rodriguez, Madelin R. Siedler, Ethan Tinoco, Grant M. Tinsley

**Affiliations:** Energy Balance & Body Composition Laboratory, Department of Kinesiology and Sport Management, Texas Tech University, Lubbock 79409, TX, USA

**Keywords:** 3D scanning, Body fat, Smartphone, Optical imaging, Digital anthropometry

## Abstract

Within the USA military, monitoring body composition is an essential component of predicting physical performance and establishing soldier readiness. The purpose of this study was to explore mobile phone three-dimensional optical imaging (3DO), a user-friendly technology capable of rapidly obtaining reliable anthropometric measurements and to determine the validity of the new Army one-site body fat equations using 3DO-derived abdominal circumference. Ninety-six participants (51 F, 45 M; age: 23·7 ± 6·5 years; BMI: 24·7 ± 4·1 kg/m^2^) were assessed using 3DO, dual-energy X-ray absorptiometry (DXA) and a 4-compartment model (4C). The validity of the Army equations using 3DO abdominal circumference was compared with 4C and DXA estimates. Compared with the 4C model, the Army equation overestimated BF% and fat mass (FM) by 1·3 ± 4·8 % and 0·9 ± 3·4 kg, respectively, while fat-free mass (FFM) was underestimated by 0·9 ± 3·4 kg (*P* < 0·01 for each). Values from DXA and Army equation were similar for BF%, FM and FFM (constant errors between −0·1 and 0·1 units; *P* ≥ 0·82 for each). In both comparisons, notable proportional bias was observed with slope coefficients of −0·08 to −0·43. Additionally, limits of agreement were 9·5–10·2 % for BF% and 6·8–7·8 kg for FM and FFM. Overall, while group-level performance of the one-site Army equation was acceptable, it exhibited notable proportional bias when compared with laboratory criterion methods and wide limits of agreement, indicating potential concerns when applied to individuals. 3DO may provide opportunities for the development of more advanced, automated digital anthropometric body fat estimation in military settings.

Body composition is an important indicator commonly used in research, healthcare and fitness settings^(1)^. Within the USA military, monitoring body composition is an essential component of predicting physical performance and establishing soldier readiness^(2)^. Maintaining soldier readiness requires the consistent assessment of aerobic fitness, anaerobic fitness and body composition to support ‘optimal well-being and performance under all conditions’^(2)^. Criterion methods of body composition estimation, such as the 4-compartment (4C) model, and laboratory methods, such as dual-energy X-ray absorptiometry (DXA), are frequently recommended for accurate body composition estimation^(3)^. However, utilisation of these methodologies is costly, and the requisite equipment may not be easily accessible; therefore, alternative techniques are often employed. For example, military assessments that aid in determining readiness of personnel require manual circumferential measurements to estimate body fat percentage (BF%). The simplicity of this methodology allows testers to assess large units of soldiers in a short period of time without the need for specialised equipment.

In the USA Army, concerns over the increasing prevalence of obesity within their ranks, as determined through BMI, have been heavily emphasised, with criticism directed at the Army Body Composition Program and its apparent lack of effectiveness^(4)^. In fact, it has been reported that ≥ 68 % of soldiers are overweight and that ≥ 21·6 % of soldiers are categorised as having obesity^(5)^. To illustrate the notable rise in these values, the obesity prevalence in August 2020 was 15 %^(6)^. In addition to the well-known health risks of obesity, associations have been observed between elevated adiposity and injury risk, another obstacle to soldier readiness^(7,8)^. The impact of obesity also extends beyond physical health and performance, with the Center for Disease and Control and Prevention estimating obesity-related medical costs to be over $1·5 billion annually for the Department of Defense, along with 658 000 lost workdays due to overweight and obesity, as established by BMI, in active-duty military personnel^(9)^. Furthermore, these estimates may not fully reflect the negative impact of lost productivity on operational readiness and national security^(10)^.

The complexity of assessing soldiers who are classified as overweight or obese begins with the potential bias exhibited by the use of BMI to establish these classifications. In fact, due to its ease of implementation, militaries around the world still rely on BMI to categorise and describe their ranks. Monitoring the effectiveness of a body composition program becomes more complicated with increases in muscle mass often experienced during military fitness training. This highlights an obvious limitation of BMI: the tendency to overestimate incidence of overweight and obesity in those with more muscle mass. Therefore, there is a present need for military forces worldwide to develop improved methodologies for feasibly assessing body composition to 1) accurately establish then monitor adiposity and health risks within their ranks and 2) facilitate a focus on increasing muscle mass and occupationally relevant performance through military physical fitness programs. The British Regular Army and USA Army, among others, have updated their fitness assessments, with the latter making substantial changes to its Army Body Composition Program^(2,11)^. In the USA, a restructuring of the Army Body Composition Program occurred following the recent completion of an Army-wide body composition investigation titled, the Army Comprehensive Body Composition (ACBC) Study. Though the results of the ACBC study have yet to be shared publicly, the Army provided an overview of the concept, design and results and has already acted on the data obtained through this investigation^(12)^. Using reference methods for BF% estimation, updated sex-specific regression equations were developed that require only two anthropometric values (abdomen circumference at the navel and body mass)^(13,14)^. The use of manually measured abdomen circumference is consistent with decades of military body composition estimation emphasising manual anthropometry^(15)^. While the low cost, portability and relative ease of use makes this method attractive, emerging digital technologies may hold promise for improving consistency in estimation across geographical sites and numerous assessors. One such technology is three-dimensional optical imaging (3DO) for digital anthropometry^(15)^.

3DO technology has become increasingly popular for quantifying body composition in the last decade^(16,17)^. The development of this assessment technique has led to the production of a spectrum of devices that allow users to estimate anthropometric and body composition variables from visual characterisations of the body. Typically, these devices collect visual data, build a three-dimensional avatar and estimate the circumferences and volumes of multiple sites on the body. In some cases, these anthropometric outputs are then used within unique algorithms to estimate body composition. Based on the well-established reliability of these technologies^(18)^, they may hold promise for standardising and digitising the collection of basic anthropometric variables used in military body composition estimation procedures. However, traditional 3DO scanners are large and relatively non-portable, potentially limiting their ease of use in diverse settings.

Within the last 5 years, there has been an increased focus on the development of mobile phone 3DO applications which simplify body scanning and increase access to digital anthropometric evaluation^(19–23)^. Currently, most mobile 3DO applications implement technology by which stationary, two-dimensional photos of the human body, typically from frontal and lateral views, are processed and then constructed into a 3D avatar for subsequent analysis^(19,20,24)^. Efforts to produce methods with the ability to capture more of the body’s surface have led to the creation of 3DO mobile phone applications that require the subject to rotate in place while the device’s camera captures visual data from all angles in less than 10 seconds. The full rotation of the subject allows 3D body avatars to be constructed using more complete data, potentially allowing for better characterisation of body shape^(22,23)^. These applications have recently demonstrated high reliability for circumferential estimates when compared with traditional, non-portable 3D scanners^(22,23)^. Accordingly, potential applications of this technology can be considered. The primary purpose of the present analysis is to explore one such application: determining the validity of the new Army one-site body fat equations in a sample population reflective of military recruits using abdomen circumference estimates obtained from a 3DO mobile phone application as compared with 4C and DXA estimates.

## Methods

### Overview

At a single research visit, adult participants were assessed using a 3DO mobile application (Prism Labs, Los Angeles, CA) producing a 3D avatar from serial images collected during a subject’s complete rotation on the flooring in front of a smartphone camera. The relevant abdominal circumference estimates were used within the recent Army one-site body fat estimation equations. Laboratory assessments of body composition were also performed to provide DXA and 4C values for comparison to the Army one-site equations.

### Participants

Individuals ≥ 18 years of age were recruited for participation. Volunteers were excluded from participating if there was a diagnosis of any disease or any medical condition that could influence body composition, if they had a history of major body altering surgery, had implanted electrical devices or were currently pregnant or breast-feeding. This study was conducted according to the guidelines laid down in the Declaration of Helsinki, and all procedures involving human subjects were approved by the Texas Tech University Institutional Review Board (IRB2022-610; date of first approval: 07/23/2022). Written informed consent was obtained from all subjects.

### Laboratory visit

Participants reported to the research laboratory after an overnight (≥ 8 h) fast from ingestion of foods, fluids and other substances, as well as a ≥ 24-hour abstention from exercise or other moderate- or vigorous-intensity physical activity. Upon arrival, each participant voided their bladder and changed into minimal, form-fitting clothing and a swim cap.

### Mobile applications

The mobile 3DO application (Prism Labs, Los Angeles, CA) required participants to rotate in place approximately 1·7 m in front of a mobile phone while serial images were captured by the camera. Scans were performed using an iPhone 13 Pro Max (model number MLKR3LL/A) with iOS v. 16.5 (Apple, Cupertino). For all scans, the iPhone was mounted on a tripod. Each scan was processed using the manufacturer’s procedures, including machine learning for data pre-processing through binary segmentation and obtaining frame-to-frame correspondences. Avatars were produced by fully non-rigid reconstruction, and a parameterised body model was fitted to each avatar to normalise the avatar’s pose to a canonical pose and promote consistent measurement locations^(22,23)^. Three scans were performed for each participant, with circumference values averaged across these scans.

**Fig. 1.**
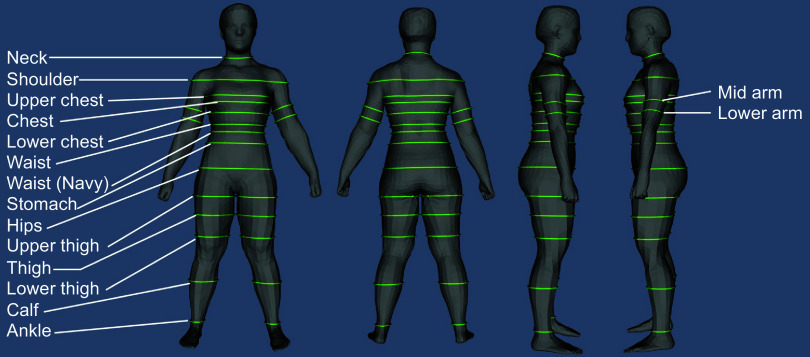
Three-dimensional avatar and circumference sites. In the present analysis, the ‘stomach’ site was selected as the abdominal circumference that approximated the navel for use in the Army one-site body fat prediction equations.

Raw values of the abdominal circumference estimates (‘stomach’; Fig. 1) obtained by the mobile phone application software were used to derive BF% estimates utilising the Army’s one-site equation. Visual inspection of avatars confirmed this circumference approximated the abdominal circumference at the level of the navel. Estimations of BF% were calculated using the sex-specific equations as dictated by the Army’s Body Composition Program^(13,14)^.

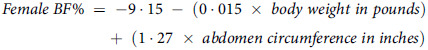




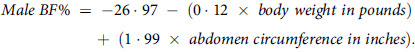




### Laboratory body composition assessments

A DXA (iDXA, General Electric, with enCORE software version 16.10.151, 16 [SP 1]) scan was performed to estimate BF% and to obtain bone mineral content for use in 4C calculations. Participants were positioned supine on the DXA table with hands neutral at their sides and feet together in a dorsiflexed position. The positioning of hands and feet was standardised using foam blocks and straps. Following the scan, body segments for analysis were distinguished with the manual adjustment of regions of interest lines using enCORE software. DXA bone mineral content was divided by 0·9582 for use in the 4C model^(25)^.

An air displacement plethysmography (ADP; BOD POD®, Cosmed USA) assessment was conducted to obtain body volume estimates for use in the 4C model. Testing was performed in accordance with manufacturer procedures in which participants wore compression clothing and a swim cap. At least two scans were performed to ensure consistent measurements. The ADP’s calibrated scale (Model BWB-627-A, modified Tanita Corp.) was used to obtain body mass.

Finally, bioimpedance spectroscopy (SFB7, ImpediMed) was performed to estimate total body water. Participants were positioned supine for at least 3 minutes with electrodes affixed 5 cm apart – two on right hand and two on the right foot. The proximal electrode of the wrist was applied between the styloid processes of the radius and ulna bones. The proximal electrode for the foot was placed between the lateral and medial malleoli.

The 4C model was produced using the equation outlined by Wang et al (2002)^(3)^.

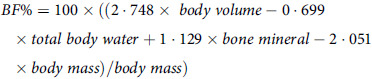




### Statistical analysis

The validity of the recent USA Army one-site body fat equation, using 3DO-dervied abdomen circumferences, was compared with 4C and DXA reference estimates. The primary outcome was BF% due to this metric being directly generated from the Army one-site equation and used for decision making in the military context (i.e. to promote external validity). In addition to the provided BF% estimate, fat mass (FM) and fat-free mass (FFM) were evaluated as additional related outcomes, as they can be directly calculated from BF% and body mass estimates. Approximately normal distributions of outcomes were confirmed using quantile–quantile plots. The linear relationships between 3DO and criterion estimates were established using ordinary least squares regression, with the reference method (4C or DXA) specified as the *x* variable and the Army equation specified as the *y* variable. Paired-samples *t* tests were performed between each reference model and the Army equation estimates. The constant error (i.e. mean difference) was calculated. Pearson’s *r* and R^2^, Lin’s concordance correlation coefficient, the root mean square error, the standard error of the estimate and mean absolute error (MAE) were also estimated. Additionally, Bland-Altman analysis was performed^(26)^, including estimation of the 95 % limits of agreement and linear regression to examine proportional bias. All statistical analyses were conducted in R (version 4.3.1), primarily using the *glmnet* (v. 4.1–7)^(27)^, *DescTools* (v. 0·99.49)^(28)^ and *deming* (v. 1.4)^(29)^ packages.

## Results

### Participants

Ninety-six participants (51 F, 45 M) were included in the present analysis (Table 1). Based on self-report, 53 participants were non-Hispanic Caucasian, 24 were Hispanic, 10 were Asian, 8 were Black and one was unspecified.

**Table 1.** Participant characteristics (Mean values and standard deviations)

		Mean	sd	Min	Max
All (*n* 96)	Age (years)	23·7	6·5	18·0	51·0
	Body Mass (kg)	71·2	14·5	42·0	116·5
	Height (cm)	169·4	9·8	150·6	194·4
	BMI (kg/m^2^)	24·7	4·1	16·9	39·5
F (*n* 51)	Age (years)	23·1	4·1	18·0	45·0
	Body Mass (kg)	63·5	10·9	42·0	89·1
	Height (cm)	162·9	6·6	150·6	180·7
	BMI (kg/m^2^)	23·9	4·1	16·9	35·3
M (*n* 45)	Age (years)	24·4	7·1	18·0	51·0
	Body Mass (kg)	80·0	13·1	56·0	116·5
	Height (cm)	176·9	7·1	165·6	194·4
	BMI (kg/m^2^)	25·6	4·0	18·0	39·5

### Evaluation of army one-site equation

**Fig. 2.**
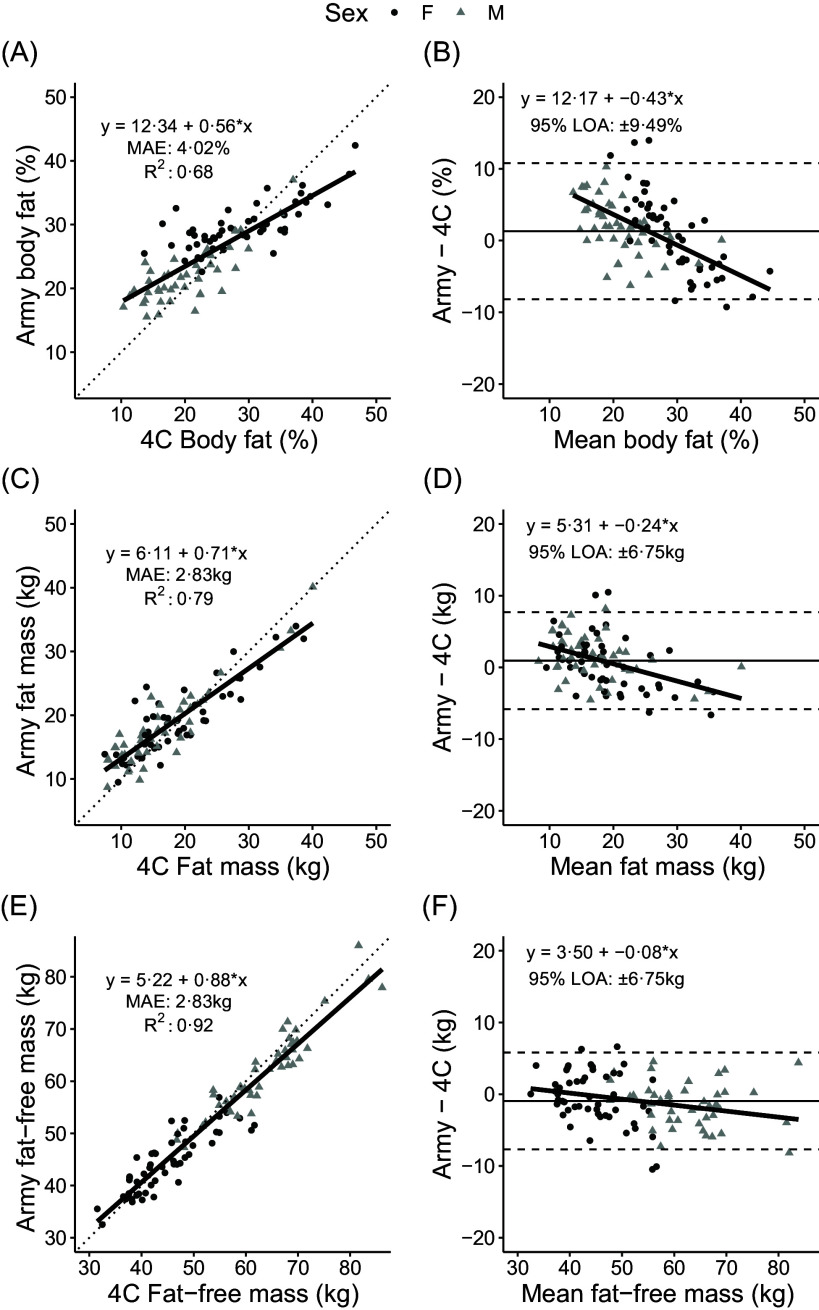
Comparison of body composition estimates obtained by the Army one-site equation using 3D scan data and a 4-compartment model. The linear relationship between 3DO and 4C values and Bland–Altman analysis are displayed for body fat % (A, B), fat mass (C, D) and fat-free mass (E, F). MAE, mean absolute error; LOA, limits of agreement.

When compared to the 4C model, BF% and FM were overestimated by 1·3 ± 4·8 % and 0·9 ± 3·4 kg, respectively, while FFM was underestimated by 0·9 ± 3·4 kg (*P* < 0·01 for each; Table 2). MAE values for the Army one-site equation were 4·0 %, 2·8 kg and 2·8 kg for BF%, FM and FFM (Fig. 2), with concordance correlation coefficient values ranging from 0·83 to 0·97. Notable proportional bias was also observed, particularly for BF%, with slope coefficients of –0·08 to –0·43. Limits of agreement were 9·5 % for BF% and 6·8 kg for FM and FFM.

**Table 2.** Validity metrics for army one-site equation (Mean values and standard deviations)

Variable	Model	Model		Army Eqn.		CE	sd	SEE	RMSE	r	CCC
		Mean	sd	Mean	sd						
BF%	4C	24·8	8·3	26·1	5·6	1·3	4·8*	3·2	5·0	0·83*	0·75*
	DXA	26·0	8·7			0·1	5·2	3·2	5·2	0·82*	0·75*
FM (kg)	4C	17·7	7·4	18·6	5·9	0·9	3·4*	2·7	3·6	0·89*	0·86*
	DXA	18·6	7·9			0·0	3·6	2·6	3·6	0·90*	0·86*
FFM (kg)	4C	53·5	12·3	52·6	11·3	–0·9	3·4*	3·2	3·6	0·96*	0·95*
	DXA	52·6	12·2			–0·1	4·0	3·7	4·0	0·95*	0·94*

Pred, predicted by newly developed equation; CE, constant error (mean difference); SEE, standard error of the estimate; RMSE, root mean square error; r: Pearson’s correlation coefficient; CCC, Lin’s concordance correlation coefficient

Results displayed for entire sample (*n* 96)

* *P* < 0·05 for paired-samples *t* test, Pearson’s correlation or Lin’s concordance correlation coefficient, as appropriate, when compared with reference model (4C or DXA).

**Fig. 3.**
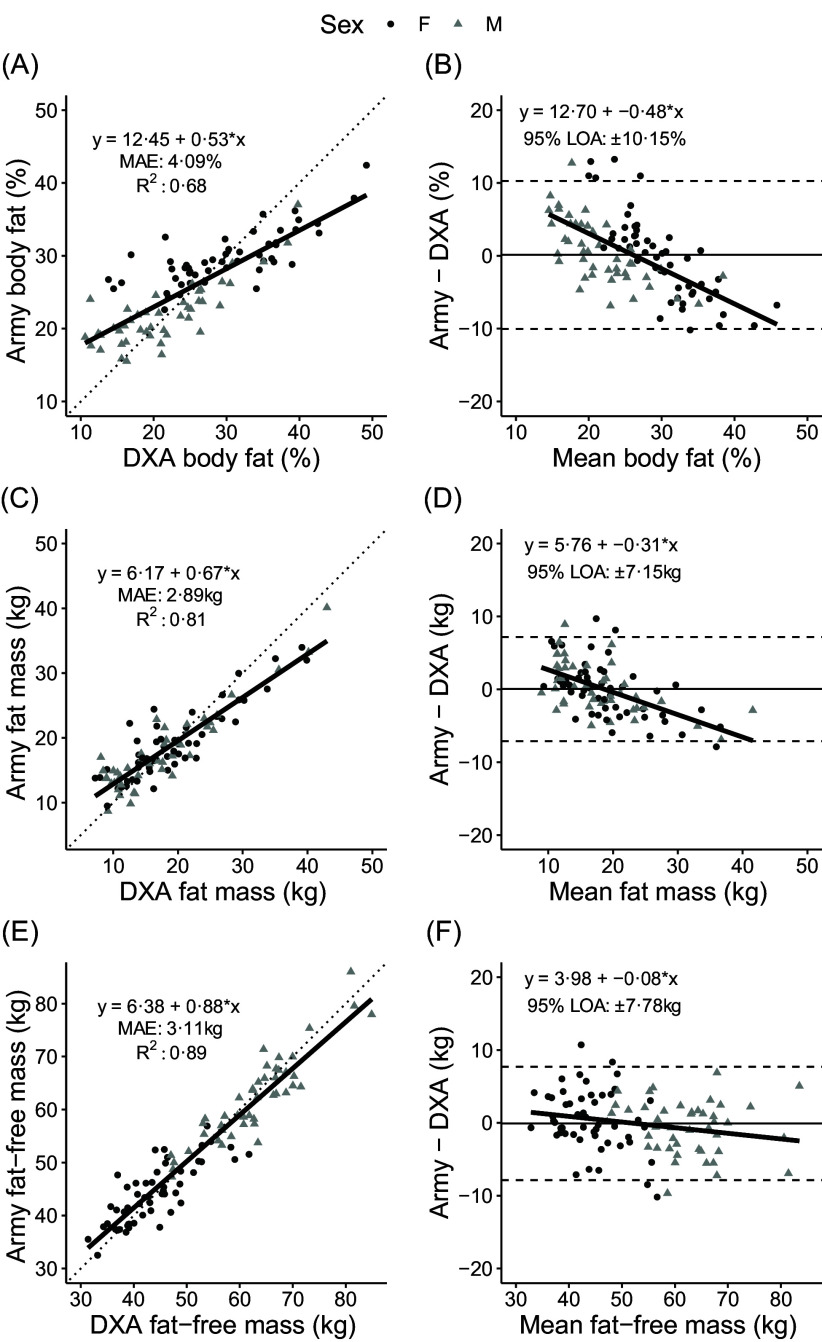
Comparison of body composition estimates obtained by the Army one-site equation using 3D scan data and dual-energy X-ray absorptiometry. The linear relationship between 3DO and 4C values and Bland–Altman analysis are displayed for body fat % (A, B), fat mass (C, D) and fat-free mass (E, F). MAE, mean absolute error; LOA, limits of agreement.

For BF%, FM and FFM, DXA and the Army one-site equation produced similar results (constant errors of 0·1 ± 5·2 %, 0·0 ± 3·6 kg and −0·1 ± 4·0 kg, respectively; *P* ≥ 0·82 for each). MAE values for the one-site USA. Army equation were 4·1 %, 2·9 kg and 3·1 kg for BF%, FM and FFM (Fig. 3), with concordance correlation coefficient values ranging from 0·78 to 0·96. Substantial proportional bias was also observed, particularly for BF%, with slope coefficients of −0·08 to −0·48. Limits of agreement were 9·5 % for BF% and 6·8 kg for FM and FFM.

## Discussion

The obesity epidemic continues to increase globally, a trend that is reflected in the USA military population. A rise in national obesity rates negatively impacts enlistment, retention, metabolic health of soldiers and operational readiness^(10,30–35)^. Beyond personnel issues, the influence of an increasingly unhealthy military force costs the nation extraordinary amounts of money in medical expenses and lost productivity. To address this ongoing concern, the Army has implemented and restructured their strategies to improve the health status of their soldiers. Among the changes, new BF% prediction equations were developed and introduced for body composition assessment. The present study investigated the validity of the new one-site Army equations based on abdomen circumference measurements acquired from a mobile scanning application using 3DO technology.

Modern standards for assessing body composition of military members were initially set forth by the Department of Defense in the 1980s with updated directives occurring in 2002 and most recently in 2022^(36)^. The Department of Defense Physical Fitness and Body Composition Program (PF/BC) directive assigns the responsibility of developing standards of physical fitness and body composition assessments to each of the military branches within overarching standards. Modifications to the PF/BC typically occur in response to a changing military population to better reflect the population. For example, the 2002 directive included regulation specifications for age and sex adjustments^(15)^. Prior to the release of the most recent directive, the topic of body composition and physical fitness as it relates to soldier readiness has been a controversial one with many investigations and reviews deeming previous programs and initiatives as ineffective^(4,6,7,10,31,37)^.

The 2022 Department of Defense PF/BC indicates that testing must be prescribed no less than once a year, and BF% thresholds are to be maintained no lower than 18 or 26 percent and no higher than 26 or 36 percent, for males and females respectively. The services were further tasked with the creation of scientifically sound prediction equations for BF% estimates with a requirement that if circumference measurements are used, the accompanying equation must be validated against established criterion methods of body composition assessment^(36)^.

The implications of suboptimal body composition can be career-ending for soldiers and costly for the military, affecting both performance and overall health. For this reason, the military requires soldiers to adhere to specific body composition standards. If a soldier is not within acceptable thresholds, he/she is ‘flagged’ and entered into the Army Body Composition Program for remedial training until the standard is met. Furthermore, during this period of remedial training, the soldier is not considered mission-ready and is ineligible for reenlistment, promotion and other opportunities. Should the soldier continue to be unable to meet standards after a period of time, a discharge from service may occur^(2)^.

Based on the notable importance placed on body composition results in the military, continued evaluation of body composition estimation methods is essential to ensure the methods used are both appropriate and feasible. In this regard, the recent ACBC study sought to gather new data on body composition estimation in more than 2600 soldiers^(12)^. Resulting adjustments to prediction equations were implemented, with new equations including abdominal circumference and body mass within sex-specific equations^(13,14)^. In the present study, we evaluated these new equations using abdominal circumferences obtained from mobile phone 3DO scanning, with comparisons to both 4C and DXA reference models. These methodologies are widely accepted as laboratory or criterion methods for body composition estimates^(38,39)^. While multi-compartment models, such as the 4C model, have advantages over individual measurement techniques, the greater availability and implementation of DXA for body composition assessment led to its inclusion in the present study to aid in comparison of the present results to other research laboratories. Regardless of reference model, the Army BF% equation produced MAE values of ∼4 % for BF%. While acceptable errors vary based on the specific application being considered, a previous study of smartphone applications for body composition estimation defined an acceptable MAE for BF% to be ≤ 3 %^(40)^; however, there is not currently a consensus in the literature. Previously used equations developed by the Navy and used by the Army produced standard error of the estimate values of ∼3·5 % for BF% in males (*n* 594) and females (*n* 202)^(41)^, very similar to the values observed for the Army one-site equation in the present study. Furthermore, when comparing the Army equation to DXA, mean BF%, FM and FFM values were very similar between methods. In contrast, an overestimation of BF% was observed when the Army equations were compared with a 4C model, which was contributed to by higher FM estimates and lower FFM estimates with the Army equation. The different group-level performance is very likely attributable to the reference method used in equation development. As DXA was one of the methods implemented in the ACBC study^(12)^ and was presumably used in the development of the new body composition equations, these results provide support for the stability of the new equations, at least at the group level, within a separate sample of young adults and when using 3DO-based abdominal circumferences.

Despite the strong group-level performance of the Army one-site equation, particularly as compared to DXA, proportional bias was observed when comparisons were made to either reference model. For BF%, notable negative proportional bias was observed, along with wide limits of agreement. Collectively, these findings indicate that there are limitations to the Army one-site equations for the individual assessment of body composition, especially in individuals with particularly low or high BF%. Further investigation into how to appropriately mitigate this proportional bias is warranted. Notably, if 3DO technology is employed to rapidly acquire anthropometric information in military settings, more advanced BF% prediction algorithms could be implemented instantaneously. While BF% prediction equations using a greater number of anthropometric inputs would increase the time and effort burden when manual anthropometry is performed, this is not the case for digital anthropometry through 3DO, for which there is no added assessment complexity when a more complex algorithm is automatically applied.

Due to its recency, the Army one-site equation has been minimally examined in previous peer-reviewed literature. However, prior investigations have quantified the performance of the earlier military body composition equations. For example, McClung and colleagues^(42)^ studied women who completed the Army Ranger course and assessed their body composition using the previous equation and DXA. They determined that the equation overestimated BF% when compared to DXA (28·6 ± 3·6 % and 20·0 ± 2·0 %, respectively). Conversely, Foulis and colleagues^(43)^ examined body composition changes in military men and women over an eight-week training period, comparing the previous equation to DXA. They observed that the equations underestimated BF% at the start of the training period for both men and women (–6·0 % ± 4·4 % and –6·0 % ± 3·5 %, respectively). Perhaps more importantly, they identified the inability of the equation to capture the changes in women’s body composition post-training, as well as an underestimation of the magnitude of changes in men. The difference in population between studies may partially explain the discrepancy between the cross-sectional findings. McClung and colleagues^(42)^ focused on elite women soldiers with a mean DXA-measured BF% of 20 % compared with Foulis and colleagues’^(43)^ population with a mean DXA-measured BF% of 32 %. Additional methodological factors, such as the specific DXA scanners, pre-assessment standardisation and individual assessors, performing manual anthropometry may have also contributed to the discrepant findings. With the implementation of the new Army one-site equations, as used in the present analysis, existing and new datasets can be examined to better establish the performance of the new equations in relevant cross-sectional and longitudinal analyses. Additionally, more complex BF% prediction equations can be explored given the ease of implementation within 3DO smartphone technology.

A unique aspect of the present analysis is the implementation of automated, 3DO-derived circumferences within the new Army one-site equation. While the Army one-site equation can already be viewed as a rapid method to estimate body composition, due to its use of a single circumference, the potential for further automation and streamlining through 3DO technology was a focus of the present study. 3DO technology for body quantification is a rapidly evolving technology. It has previously exhibited acceptable validity for some anthropometry or body composition applications when compared with well-accepted reference models^(24,40,44–47)^. Moreover, the development of 3DO mobile applications has facilitated a time-efficient and easy-to-use method of acquiring anthropometric data. In a military setting, the potential use of 3DO mobile scans promotes interrater reliability in circumferential measurements and the objective standardisation of body locations. The reliability previously demonstrated by the 3DO mobile application^(23)^ may offer improved monitoring of circumferences over a period of time, particularly when replacing multiple manual assessors. However, it could be argued that relying on a digital imaging technology for assessment of circumferences that are readily obtainable via flexible tape measure is an unnecessary complication. Further study is also necessary into whether this technology can be implemented to accurately assess longitudinal body composition changes when employed within prediction equations^(15)^. Additionally, it should be noted that other accessible technologies are available that could hold relevance for body composition assessment in military contexts. Of note is bioelectrical impedance analysis, which spans a wide range of analysers, from inexpensive consumer models to rigorously validated medical-grade devices^(48,49)^. While this technique has demonstrated tremendous evolution over the past decades, one potential challenge to implementation in military settings is the greater influence of pre-assessment procedures, such as food intake, fluid ingestion and exercise, as compared with anthropometric technologies, including 3DO^(50)^.

As discussed, the present findings indicate the potential need for continued refinement of body composition estimation methods within military settings. Trade-offs between accuracy and feasibility should be considered, with recent technological advances offering potential solutions that may improve reliability of body composition estimates with minimal logistical constraints. A limitation of the present analysis is the relatively small sample, which limits the strength of conclusions that can be drawn about equation performance and the ability to stratify performance based on demographic factors like sex and race or ethnicity. As such, further external cross-validation in larger samples is necessary. Notably, existing datasets may be explored for these purposes, provided that they contain the relevant anthropometric data (e.g. an appropriate abdominal circumference) and reference body composition estimates in military or military-relevant populations. An additional consideration is whether the 3DO scanning procedures can be appropriately implemented to produce high-quality data, namely through proper preparation of individuals being evaluated (e.g. clothing requirements and pre-assessment standardisation) and through implementation of the technology itself by multiple assessors across sites. However, the simplicity of operating the application interface is expected to be a benefit to implementation in this regard.

In summary, while the group-level performance of the one-site Army equation in the present study was acceptable, it exhibited substantial proportional bias when compared to two laboratory criterion methods. As body composition results in military contexts are used for individual-level decisions, the implications of these findings should be considered. This investigation also provides proof-of-concept support for utilising mobile phone 3DO circumference estimates within anthropometric body composition prediction equations. The potential of this technology to accessibly enhance the application of circumference-based body composition equations should continue to be investigated in future work, including a consideration of more advanced body composition prediction equations based on multiple digital anthropometric inputs.

## References

[1] Santanasto AJ , Goodpaster BH , Kritchevsky SB , et al. (2017) Body composition remodeling and mortality: the health aging and body composition study. J Gerontol A Biol Sci Med Sci 72, 513–519. 27567109 10.1093/gerona/glw163 PMC5897837

[2] U.S. Department of the Army (2019) Army Regulation 600–9: The Army Body Composition Program. –https://armypubs.army.mil/epubs/DR_pubs/DR_a/pdf/web/ARN7779_AR600–9_FINAL.pdf (accessed April 2024).

[3] Wang Z , Pi-Sunyer FX , Kotler DP , et al. (2002) Multicomponent methods: evaluation of new and traditional soft tissue mineral models by *i*n vivo neutron activation analysis. Am J Clin Nutr 76, 968–974. 12399267 10.1093/ajcn/76.5.968

[4] Meyer S & Cole R (2019) Army body composition program study results concerning: enrollees are more over fat than expected. Mil Med 184, 400–408. 30901401 10.1093/milmed/usy302

[5] American Security Project (2023) Combating Military Obesity: Stigma’s Persistent Impact on Operational Readiness. https://s3.documentcloud.org/documents/24040523/ref-0286-combating-military-obesity-2.pdf (accessed April 2024).

[6] Gravina D , Keeler JL , Akkese MN , et al. (2023) Randomized controlled trials to treat obesity in military populations: a systematic review and meta-analysis. Nutrients 15, 4778. 38004172 10.3390/nu15224778 PMC10674729

[7] Knapik JJ , Farina EK , Steelman RA , et al. (2023) The medical burden of obesity and overweight in the US Military: association of BMI with clinically diagnosed medical conditions in United States Military Service Members. J Nutr 153, 2951–2967. 37619919 10.1016/j.tjnut.2023.08.023

[8] Reyes-Guzman CM , Bray RM , Forman-Hoffman VL , et al. (2015) Overweight and obesity trends among active duty military personnel: a 13-year perspective. Am J Prev Med 48, 145–153. 25442226 10.1016/j.amepre.2014.08.033

[9] Centers for Disease Control and Prevention (2023) Unfit to Serve: Obesity and Physical Activity are Impacting National Security. –https://www.cdc.gov/physicalactivity/downloads/unfit-to-serve-062322–508.pdf (accessed April 2024).

[10] Dall TM , Zhang Y , Chen YJ , et al. (2007) Cost associated with being overweight and with obesity, high alcohol consumption, and tobacco use within the military health system’s TRICARE prime-enrolled population. Am J Health Promot 22, 120–139. 18019889 10.4278/0890-1171-22.2.120

[11] The British Army (2024) Fit to Fight: The New Role Fitness Test Entry. https://www.army.mod.uk/media/8240/fit-to-fight-the-new-role-fitness-test-e.pdf (accessed April 2024).

[12] Phillips C (2023) The Science Behind the Army Comprehensive Body Composition Study: USARIEM Completes Critical Data Collection. https://usariem.health.mil/index.cfm/media/news/article/2023/the_science_behind_the_army_comprehensive_body_composition_study (accessed April 2024).

[13] United States Army (2023) DA Form 5501, Body Fat Content Worksheet (Female). https://recruiting.army.mil/Portals/15/DA5501.pdf (accessed April 2024).

[14] United States Army (2023) DA Form 5500, Body Fat Content Worksheet (Male). https://recruiting.army.mil/Portals/15/DA5500.pdf (accessed April 2024).

[15] Harty PS , Friedl KE , Nindl BC , et al. (2022) Military body composition standards and physical performance: historical perspectives and future directions. J Strength Cond Res 36, 3551–3561. 10.1519/JSC.0000000000004142 34593729

[16] Heymsfield SB , Bourgeois B , Ng BK , et al. (2018) Digital anthropometry: a critical review. Eur J Clin Nutr 72, 680–687. 29748657 10.1038/s41430-018-0145-7 PMC6411053

[17] Mocini E , Cammarota C , Frigerio F , et al. (2023) Digital anthropometry: a systematic review on precision, reliability and accuracy of most popular existing technologies. Nutrients 15, 302. 36678173 10.3390/nu15020302 PMC9864001

[18] Tinsley GM , Moore ML , Dellinger JR , et al. (2020) Digital anthropometry via three-dimensional optical scanning: evaluation of four commercially available systems. Eur J Clin Nutr 74, 1054–1064. 31685968 10.1038/s41430-019-0526-6

[19] Nana A , Staynor JMD , Arlai S , et al. (2022) Agreement of anthropometric and body composition measures predicted from 2D smartphone images and body impedance scales with criterion methods. Obes Res Clin Pract 16, 37–43. 35094958 10.1016/j.orcp.2021.12.006

[20] Farina GL , Orlandi C , Lukaski H , et al. (2022) Digital single-image smartphone assessment of total body fat and abdominal fat using machine learning. Sensors (Basel) 22, 8365. 36366063 10.3390/s22218365 PMC9657201

[21] McCarthy C , Tinsley GM , Yang S , et al. (2023) Smartphone prediction of skeletal muscle mass: model development and validation in adults. Am J Clin Nutr 117, 794–801. 36822238 10.1016/j.ajcnut.2023.02.003 PMC10315403

[22] Tinsley GM , Harty PS , Siedler MR , et al. (2023) Improved precision of 3-dimensional optical imaging for anthropometric measurement using non-rigid avatar reconstruction and parameterized body model fitting. Clin Nutr Open Sci 50, 40–45.

[23] Tinsley GM , Rodriguez C , Siedler MR , et al. (2024) Mobile phone applications for 3-dimensional scanning and digital anthropometry: a precision comparison with traditional scanners. Eur J Clin Nutr 78, 509–514. 10.1038/s41430-024-01424-w 38454153

[24] Graybeal AJ , Brandner CF & Tinsley GM (2023) Validity and reliability of a mobile digital imaging analysis trained by a four-compartment model. J Hum Nutr Diet 36, 905–911. 36451080 10.1111/jhn.13113 PMC10198803

[25] Tinsley GM (2021) Five-component model validation of reference, laboratory and field methods of body composition assessment. Br J Nutr 125, 1246–1259. 32921319 10.1017/S0007114520003578

[26] Bland JM & Altman DG (1986) Statistical methods for assessing agreement between two methods of clinical measurement. Lancet 1, 307–310. 2868172

[27] Friedman JH , Hastie T & Tibshirani R (2010) Regularization paths for generalized linear models via coordinate descent. Journal of Statistical Software 33, 1–22. 20808728 PMC2929880

[28] Signorell A (2022) DescTools: Tools for Descriptive Statistics. https://cran.r-project.org/package=DescTools (accessed April 2024).

[29] Therneau T (2018) deming: Deming, Theil-Sen, Passing-Bablock and Total Least Squares Regression. https://CRAN.R-project.org/package=deming (accessed April 2024).

[30] Cowan DN , Bedno SA , Urban N , et al. (2011) Musculoskeletal injuries among overweight army trainees: incidence and health care utilization. Occup Med 61, 247–252. 10.1093/occmed/kqr028 21482621

[31] Bornstein DB , Grieve GL , Clennin MN , et al. (2019) Which US states pose the greatest threats to military readiness and public health? Public health policy implications for a cross-sectional investigation of cardiorespiratory fitness, body mass index, and injuries among US Army recruits. J Public Health Manag Pract 25, 36–44. 29319585 10.1097/PHH.0000000000000778

[32] Jones BH , Hauret KG , Dye SK , et al. (2017) Impact of physical fitness and body composition on injury risk among active young adults: a study of Army trainees. J Sci Med Sport 20, S17–S22. 28993131 10.1016/j.jsams.2017.09.015

[33] Nye NS , Kafer DS , Olsen C , et al. (2018) Abdominal circumference *v.* body mass index as predictors of lower extremity overuse injury risk. J Phys Act Health 15, 127–134. 28872394 10.1123/jpah.2017-0017

[34] Knapik JJ , Sharp MA , Canham-Chervak M , et al. (2001) Risk factors for training-related injuries among men and women in basic combat training. Med Sci Sports Exerc 33, 946–954. 11404660 10.1097/00005768-200106000-00014

[35] Pierce JR , DeGroot DW , Grier TL , et al. (2017) Body mass index predicts selected physical fitness attributes but is not associated with performance on military relevant tasks in U.S. Army Soldiers. J Sci Med Sport 20, S79–S84. 10.1016/j.jsams.2017.08.021 28919497

[36] Under Secretary of Defense for Personnel and Readiness (2022) DoDI 1038.03: DoD Physical Fitness/Body Composition Program. https://www.esd.whs.mil/Portals/54/Documents/DD/issuances/dodi/130803p.pdf?ver=v5atpuD4j_nbloEbongDAA%3D%3D (accessed April 2024).

[37] Nelson R , Cheatham J , Gallagher D , et al. (2019) Revisiting the United States Army body composition standards: a receiver operating characteristic analysis. Int J Obes (Lond) 43, 1508–1515. 30181655 10.1038/s41366-018-0195-x

[38] Heymsfield SB , Ebbeling CB , Zheng J , et al. (2015) Multi-component molecular-level body composition reference methods: evolving concepts and future directions. Obes Rev 16, 282–294. 25645009 10.1111/obr.12261 PMC4464774

[39] Shepherd JA , Ng BK , Sommer MJ , et al. (2017) Body composition by DXA. Bone 104, 101–105. 28625918 10.1016/j.bone.2017.06.010 PMC5659281

[40] Majmudar MD , Chandra S , Yakkala K , et al. (2022) Smartphone camera based assessment of adiposity: a validation study. NPJ Digit Med 5, 79. 35768575 10.1038/s41746-022-00628-3 PMC9243018

[41] Hodgdon J & Friedl K (1999) Development of the DoD Body Composition Estimation Equations. https://apps.dtic.mil/sti/tr/pdf/ADA370158.pdf (accessed April 2024).

[42] McClung HL , Spiering BA , Bartlett PM , et al. (2022) Physical and physiological characterization of female elite warfighters. Med Sci Sports Exerc 54, 1527–1533. 35621397 10.1249/MSS.0000000000002942 PMC9390221

[43] Foulis SA , Friedl KE , Spiering BA , et al. (2023) Body composition changes during 8 weeks of military training are not accurately captured by circumference-based assessments. Front Physiol 14, 1183836. 37351259 10.3389/fphys.2023.1183836 PMC10282178

[44] Smith B , McCarthy C , Dechenaud ME , et al. (2022) Anthropometric evaluation of a 3D scanning mobile application. Obesity (Silver Spring) 30, 1181–1188. 35491718 10.1002/oby.23434 PMC9177647

[45] Graybeal AJ , Brandner CF & Tinsley GM (2023) Evaluation of automated anthropometrics produced by smartphone-based machine learning: a comparison with traditional anthropometric assessments. Br J Nutr 130, 1077–1087. 36632007 10.1017/S0007114523000090 PMC10442791

[46] Harty PS , Sieglinger B , Heymsfield SB , et al. (2020) Novel body fat estimation using machine learning and 3-dimensional optical imaging. Eur J Clin Nutr 74, 842–845. 32203233 10.1038/s41430-020-0603-x PMC7220828

[47] Minetto MA , Pietrobelli A , Busso C , et al. (2022) Digital anthropometry for body circumference measurements: European phenotypic variations throughout the decades. J Pers Med 12. 10.3390/jpm12060906 PMC9224732 35743690

[48] Siedler MR , Rodriguez C , Stratton MT , et al. (2023) Assessing the reliability and cross-sectional and longitudinal validity of fifteen bioelectrical impedance analysis devices. Br J Nutr 130, 827–840. 36404739 10.1017/S0007114522003749 PMC10404482

[49] Bosy-Westphal A , Schautz B , Later W , et al. (2013) What makes a BIA equation unique? Validity of eight-electrode multifrequency BIA to estimate body composition in a healthy adult population. Eur J Clin Nutr 67, S14–S21. 23299866 10.1038/ejcn.2012.160

[50] Tinsley GM , Harty PS , Stratton MT , et al. (2022) Tracking changes in body composition: comparison of methods and influence of pre-assessment standardisation. Br J Nutr 127, 1656–1674. 34325758 10.1017/S0007114521002579

